# **Coronary artery calcification in patients with advanced chronic kidney disease**

**DOI:** 10.1186/s12872-022-02879-0

**Published:** 2022-10-29

**Authors:** Xiadan Xiang, Ji He, Wei Zhang, Qiang He, Yueming Liu

**Affiliations:** 1 Department of Nephrology, the Second affiliated Hospital of Zhejiang Chinese Medical University, Hangzhou, Zhejiang China; 2 Urology & Nephrology Center, Department of Nephrology, Affiliated People’s Hospital, Zhejiang Provincial People’s Hospital, Hangzhou Medical College, Hangzhou, Zhejiang China

**Keywords:** Chronic kidney disease, Glomerulonephritis, Vascular calcification

## Abstract

**Introduction:**

Cardiovascular disease (CVD) is associated with higher morbidity and mortality rates in patients with chronic kidney disease (CKD). Studies have shown that vascular calcification is a major predictor of CVD. Vascular calcification in the CKD population is associated with various risk factors, and changes in bone and mineral metabolism have been linked to an increased risk of atherosclerosis. Therefore, we aimed to investigate the correlation between vascular calcification and bone metabolism, which is necessary to improve the survival and prognosis of patients with CKD.

**Methods:**

We included 146 patients with CKD who received coronary artery calcification (CAC) scores at our hospital from May 2017 to November 2018. Spearman rank correlation analysis, Mann–Whitney U test, and Kaplan–Meier method were used to analyze laboratory data and all-cause mortality.

**Results:**

In the 146 patients, chronic glomerulonephritis accounted for the most common cause of CKD, at approximately 39.0%. Spearman rank correlation analysis on the factors influencing vascular calcification in patients with CKD showed that CAC score was significantly and positively correlated with C-reactive protein, N-terminal/midregion osteocalcin (N-MID), N-terminal peptide of type 1 procollagen (P1NP), β-cross-linked C-telopeptide of type 1 collagen (β-CTx), and parathyroid hormone (*P* = 0.0423, *P* = 0.0432, *P* = 0.0235, *P* = 0.0061, *P* < 0.0001, respectively). Serum calcium levels were positively correlated with N-MID, P1NP, β-CTx, and iPTH (*r* = 0.19, *r* = 0.24, *r* = 0.21, *r* = 0.21, respectively), and serum phosphorus levels were positively correlated with N-MID, P1NP, β-CTx, and iPTH (*r* = 0.50, *r* = 0.37, *r* = 0.50, *r* = 0.55, respectively). However, no difference was found in CVC scores among patients with CKD in different stages and receiving different treatments. In the Kaplan–Meier analysis of all-cause hospitalization and mortality rates, patients with CAC > 400 had a higher risk.

**Conclusion:**

We found that the primary cause of CKD is glomerulonephritis, and the CAC score is positively correlated with inflammatory and bone metabolism markers, with a higher risk of all-cause mortality and cardiovascular hospitalization when the CAC score is greater than 400.

## Introduction

Chronic kidney disease (CKD) is a major public health concern worldwide [1]. The overall prevalence of CKD in China is approximately 10.8%, affecting an estimated population of 120 million [2]. Patients with CKD or end-stage renal disease have a significantly higher incidence and mortality of cardiovascular disease (CVD) than the general population [3]. Vascular calcification is a pathological process characterized by the accumulation of mineral deposits on arterial walls and is considered a major predictor of CVD development [4]. It is a common complication in patients with CKD, which often occurs alongside a multitude of adverse cardiac events, including arrhythmias, ischemic damage, myocardial infarction, and sudden cardiac death [5]. Early diagnosis and intervention for vascular calcification may prevent the progression of CVD in patients with CKD.

The pathological mechanisms of vascular calcification in the population with CKD are complex and involve traditional risk factors, including sex, age, smoking history, hypertension, diabetes, and hyperlipidemia, as well as risk factors unique to patients with CKD, such as kidney function decline, elevated parathyroid hormone (PTH) level, inflammation, oxidative stress, and mineral and bone disorders [6]. (In CKD, a cell-mediated process driven by alterations in vascular smooth muscle cells called tunica media vascular calcification is a characteristic manifestation of EVA [7, 8]. The milieu created by uremic toxins and other factors that induce cellular oxidative stress is key to VSMCs calcification[9–11]. Hypercalcemia and hyperphosphatemia will disrupt the inhibitory defense system in CKD, while hyperparathyroidism and hypomagnesemia have the potential to be further aggravated [9, 12]. Imbalanced signal transmission if not promptly adjusted, activation of the necessary osteogenic / chondrogenic transcription factors in the context of high calcium and phosphate will further induce VSMC osteogenic / chondrogenic protein expression. Ultimately, uremic VSMCs undergo osteochondrogenic differentiation and vascular ossification[13–16]).Previous evidence revealed that hyperphosphatemia and hypercalcemia are associated with increased cardiovascular mortality in patients with CKD [17]. Additionally, a meta-analysis consisting of 25 studies and 10,299 patients suggested that patients with altered bone and mineral metabolism should be screened for the risk of atherosclerotic vascular abnormalities [18]. ( the literature prevalence of CACs at the moment of transplantation between 35% and 70% is reported[19, 20]; According to reports by Alfier [21]et al the presence of CACs at T0 and the age were the only independent factors in determining the CAC progression )Therefore, a better understanding of the correlation between vascular calcification and bone metabolism may contribute to the development of novel management strategies for mineral and bone disorders, thereby improving the survival and prognosis of patients with CKD.

In the current study, we aimed to investigate the correlation between coronary artery calcification (CAC) score and bone metabolism indices in patients with CKD. The clinical outcomes after the CAC test were also evaluated. Our results showed that patients with CKD with a high risk of vascular calcification also showed an increased rate of all-cause and cardiovascular hospitalizations.

## Materials and methods

### Subjects

A total of 146 patients diagnosed with CKD, who underwent CAC scoring at our hospital between May 2017 and November 2018, were recruited for this study. The diagnosis and staging of CKD were based on the Kidney Disease: Improving Global Outcomes (KDIGO) clinical practice guideline for the evaluation and management of chronic kidney disease. CKD is defined as kidney damage or an estimated glomerular filtration rate (eGFR) of < 60 ml/min/1.73 m^2^ for more than 3 months. eGFR was calculated according to diet modification in renal disease study equation: eGFR = 186 × (serum creatinine)^−1.154^ × (age)^−0.203^ × (0.742, if female). Patients with severe liver diseases, malignant tumors, or acute inflammatory diseases were excluded.

CAC was determined using spiral computed tomography (CT) as previously described [22]. CT was performed by an experienced radiologist using a 640-slice CT scanner (Toshiba, Tokyo, Japan). CAC was scored according to the Agatston scoring method provided by the KDIGO, in which the lesion score was calculated by multiplying the lesion area (mm^2^) by a density factor [23]. Weighted density scores were graded as follows: 130–199 HU: 1, 200–299 HU: 2, 300–399 HU: 3, and ≥ 400 HU: 4. The total CAC score was calculated by summing the scores for all lesions. A score of 0 was defined as no risk of cardiovascular events or mortality; 1–100, low risk; 101–400, intermediate risk; and > 400, high risk [24].

### Clinical and demographic data collection

The clinical and demographic profiles of all patients were collected. Fasting blood samples were collected from all subjects for the detection of hemoglobin (HGB), C-reactive protein (CRP), albumin (ALB), creatinine, blood urea nitrogen, calcium (Ca), phosphorus (P), fasting blood glucose (FBG), PTH, 25 hydroxyvitamin D (25(OH)D), N-terminal peptide of type 1 procollagen (P1NP), β-cross-linked C-telopeptide of type 1 collagen (β-CTx), and N-terminal/midregion osteocalcin (N-MID) using AU5821 Clinical Chemistry Analyzer (Beckman Coulter, Inc., Brea, USA), XE-2100 Hematology Analyzer (Sysmex, Kobe, Japan), and Cobas E601 Immunology Analyzer (Roche, Basel, Switzerland). When the serum ALB level was less than 40 g/L, the measured serum Ca was corrected for ALB concentration (g/L) as follows: corrected Ca (mmol/L) = measured Ca (mmol/L) + 0.02 × (40-ALB concentration). The IE33 echocardiography system (Philips, Amsterdam, Netherlands) was used to measure the indicators of cardiac structure and function, including the left atrial diameter, right atrial diameter, left ventricular (LV) diameter, right ventricular diameter, and LV ejection fraction.

### Hospitalization and survival analysis

The hospitalization and mortality rates of the patients between the administration of the CAC test and the day of data collection (or death) were recorded. One patient was lost to follow-up after CAC testing. Echocardiographic results from three patients were missing. Therefore, a total of 146 patients were included in the analysis. All-cause mortality rate was defined as the percentage of deaths among the 146 patients. Major adverse cardiovascular events (a single or composite endpoint of myocardial infarction, stroke, heart failure, or cardiovascular death) were also recorded [25].

### Statistical analysis

The program R3.01 was used for data analysis and plotting of graphs. Quantitative variables with non-normal distributions are expressed as medians (Q1, Q3) and analyzed using Spearman’s rank correlation analysis. Qualitative data are summarized as percentages (%). The Mann–Whitney U test was used to compare statistical significance among groups. The Kaplan–Meier method was used for survival analysis. Statistical significance was set at *P* < 0.05.

## Results

### Clinical and demographic characteristics of patients with CKD

A total of 146 patients with CKD with a mean age of 60.49 ± 16.39 years were included in the analysis. The patients comprised 92 men (63%) and 54 women (37%). The most common causes of CKD identified in this population were chronic glomerulonephritis (57, 39.0%), diabetic nephropathy (41, 28.1%), and hypertensive nephropathy (34, 23.3%). The etiologies of CKD in the remaining 14 patients (9.6%) were kidney obstruction and drugs toxic to the kidney (Table 1). The median levels of HGB, CRP, and ALB in these patients were 96.5 (84.75, 109.25) g/L, 7.80 (1.30, 25.18) mg/L, and 34.04 ± 4.96 g/L, respectively. According to the Clinical Practice Guidelines for Chronic Kidney Disease-Mineral and Bone Disorder (CKD-MBD) provided by the KDIGO [26], 37.35%, 44.58%, and 78.31% of the patients showed abnormalities in Ca, P, and PTH levels, respectively. The echocardiographic results showed that the median left atrial diameter, right atrial diameter, and LV ejection fraction were 39 (35, 43) mm, 22 (20, 23) mm, and 63 (57, 67) %, respectively.

### Vascular calcification in patients with CKD

The median CAC score in 146 patients with CKD was 216 (1.00, 909.75) (Table 1). Thirty-five (23.97%) patients had a CAC score of 0. Twenty-six (17.81%) patients were categorized into the low-risk group, with a score of 1–100. Twenty-nine (19.86) patients were in the intermediate-risk group, with a score of 101–400. Fifty-six (38.36%) patients had a CAC score > 400 (high-risk group). In total, 85 patients had a CAC score > 100, indicating that the prevalence of vascular calcification in the study participants was 58.22%.

**Table 1:** Baseline characteristics

	Overall (n = 146)
**Age (yr, mean ± SD)**	60.49 ± 16.39
**Sex, n (%)**	
male	92 (63.0%)
female	54 (37.0%)
**Primary renal disease, n (%)**	
CGN	57 (39.0%)
DN	41 (28.1%)
HTN	34 (23.3%)
Others	14 (9.6%)
**RRT, n (%)**	
HD	83 (56.8%)
PD	37 (25.3%)
no	26 (17.8%)
**Laboratory analysis**	
HGB	96.5 (84.75, 109.25)
CRP	7.80 (1.30, 25.18)
ALB	34.04 ± 4.96
Ca	2.35 ± 0.22
P	1.68 (1.26, 2.27)
**Bone metabolic markers**	
iPTH	284.35 (108.55, 983.73)
βCTx	1861.50 (1133.25, 4610.75)
N-MID	176.85 (59.90, 275.03)
25[OH]D	13.80 (7.55, 22.52)
P1NP	239.90 (139.53, 1200.00)
**Cardiac ultrasound**	
LA (mm)	38.91 ± 6.32
RV (mm)	22.00 (20.00, 23.00)
EF (%)	63 (57, 67)
**Calcification score M(Q1,Q3)**	216.00 (1.00, 909.75)
**n(%)**	
0	35 (23.97%)
1-100	26 (17.81%)
101–400	29 (19.86%)
> 400	56 (38.36%)

*CGN: Chronic glomerulonephritis; DN: Diabetic nephropathy; HTN: Hypertensive nephropathy; HD: Hemodialysis; PD: Peritoneal dialysis; iPTH: parathyroid hormone; βCTx: β cross-linked C telopeptide of type 1 collagen; N-MID:N-MID osteocalcin; 25[OH]D:25 hydroxyl-vitamin D; P1NP:N-terminal peptide of type 1 procollagen; LA :Left atrial diameter; RV: Right ventricular diamete; EF: Ejection fraction.

### Factors influencing vascular calcification in patients with CKD

**Fig. 1:**
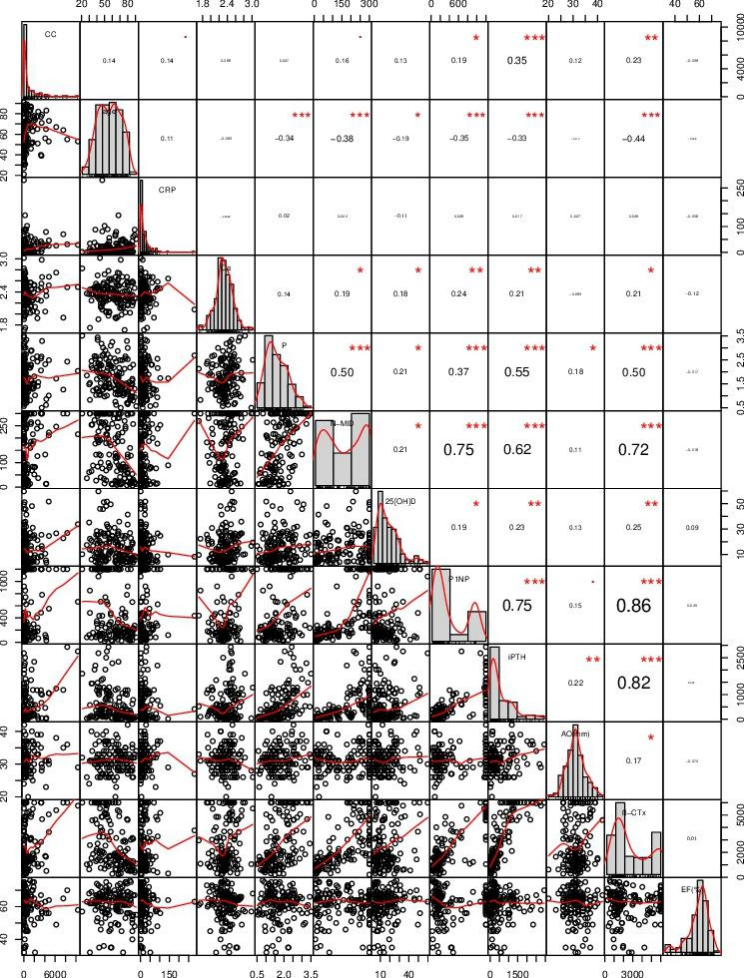
**Factors influencing vascular calcification in CKD patients** (Spearman rank correlation analysis was performed to identify the factors influencing vascular calcification in CKD patients. * indicates statistical significance. * *P* < 0.05, ** *P* < 0.01, *** *P* < 0.001. CRP, c-reactive protein; Ca, calcium; P, phosphorus; N-MID, N-MID osteocalcin, 25(OH)D, 25 hydroxyl-vitamin D; P1NP, N-terminal peptide of type 1 procollagen; PTH, parathyroid hormone; AO, aorta diameter; β-CTx, β cross-linked C-telopeptide of type 1 collagen; EF (%), left ventricular ejection fraction.)

Spearman’s rank correlation analysis was performed to identify the factors influencing vascular calcification in these patients. The results showed that CAC score was not correlated with age, Ca, P, 25(OH)D, or LV ejection fraction (*P* = 0.1028, *P* = 0.4299, *P* = 0.7441, *P* = 0.1232, *P* = 0.6446, respectively). In addition, no significant correlations were detected in CAC score and HGB, ALB, creatinine, blood urea nitrogen, and FBG levels (data not shown). However, CAC score was significantly and positively correlated with CRP, N-MID, P1NP, β-CTx, and PTH levels (*P* = 0.0423, *r* = 0.14; *P* = 0.0432, *r* = 0.16; *P* = 0.0235, *r* = 0.19; *P* = 0.0061, *r* = 0.23; *P* < 0.0001, *r* = 0.35, respectively). Serum Ca level was positively and significantly correlated with N-MID, P1NP, β-CTx, and iPTH levels (*r* = 0.19, *r* = 0.24, *r* = 0.21, *r* = 0.21, respectively), and serum P level was positively correlated with N-MID, P1NP, β-CTx, and iPTH (*r* = 0.50, *r* = 0.37, *r* = 0.50, *r* = 0.55, respectively) with statistical significance (Fig. 1).

### Vascular calcification in patients with CKD at different stages

**Fig. 2:**
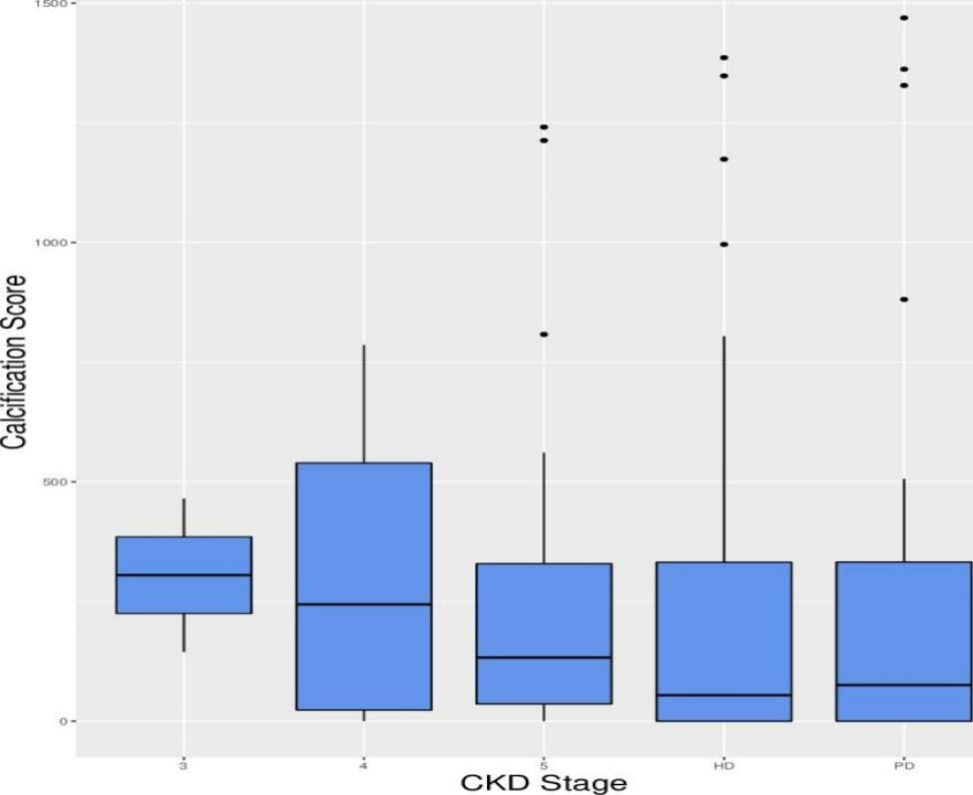
**CAC scores in CKD patients receiving different treatments** (CKD patients were categorized into five groups according to their eGFR: CKD stage 3, CKD stage 4, CKD stage 5 (without dialysis), CKD stage 5 (hemodialysis), and CKD stage 5 (peritoneal dialysis). Box plots were generated to demonstrate the show the overall distribution of CAC scores in each group.)

Patients with CKD were categorized into the following groups based on their eGFR: CKD stage 3, CKD stage 4, CKD stage 5 (without dialysis), CKD stage 5 (hemodialysis), and CKD stage 5 (peritoneal dialysis). There were 2 (1.4%), 4 (2.7%), 20 (13.7%), 83 (56.8%), and 37 (25.3%) patients in the above-mentioned groups with CAC scores of 305 (225, 385), 244 (23.25, 539.25), 182 (51, 909.25), 225 (1, 900), and 224 (0, 1328), respectively (Table 2; Fig. 2). No significant difference was found in the CAC scores among these groups (*P* = 0.985).

**Table 2:** Prevalence of vascular calcification in CKD patients at different stages

CKD stage	Cases n (%)	CAC score M(Q1,Q3)
3	2 (1.4%)	305 (225, 385)
4	4 (2.7%)	244 (23.25, 539.25)
5*	20 (13.7%)	182 (51, 909.25)
-HD	83 (56.8%)	225 (1, 900)
-PD	37 (25.3%)	224 (0, 1328)

*No dialysis treatment

### Hospitalization and survival analysis

**Fig. 3:**
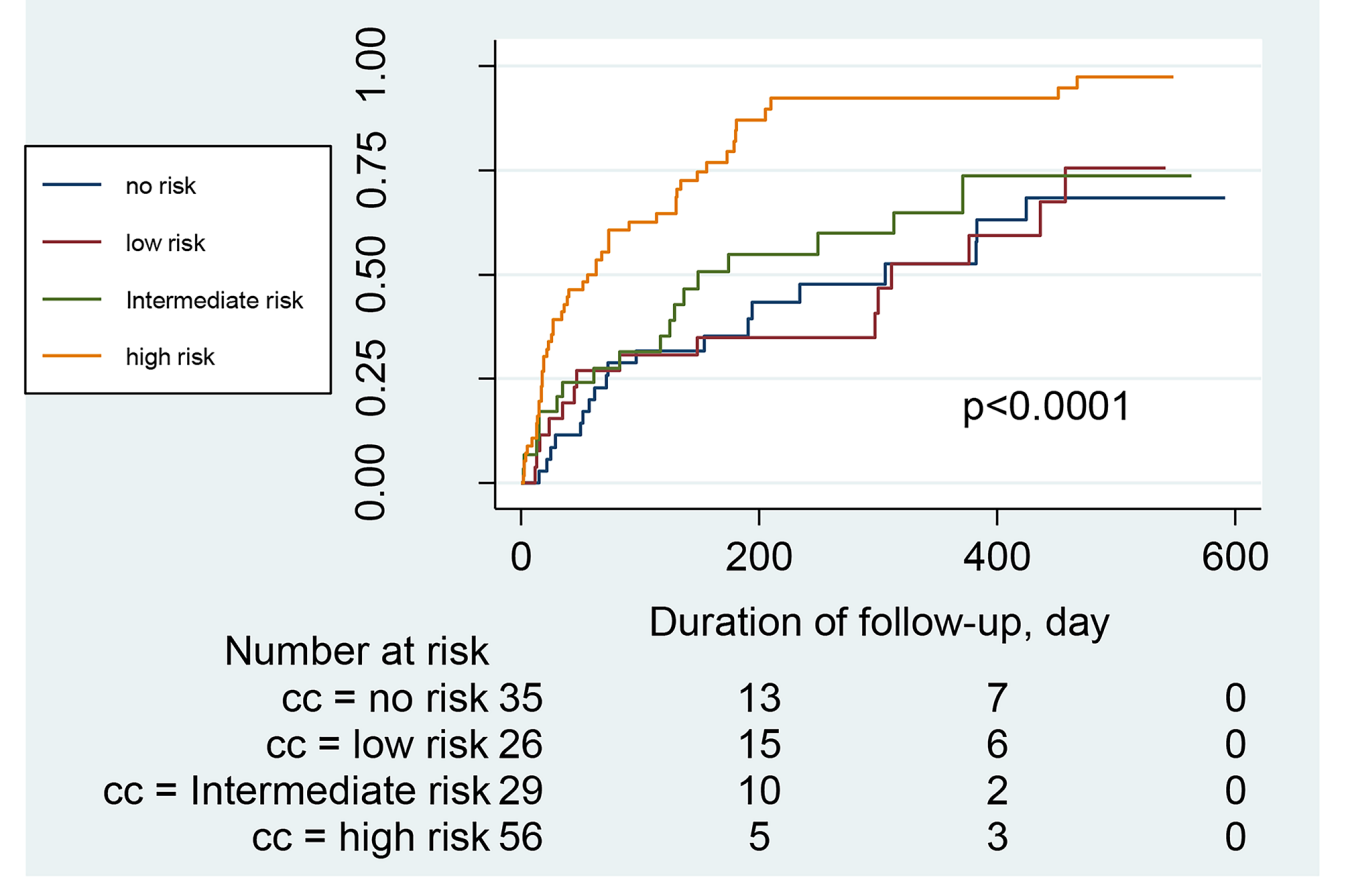
**Survival curves for all-cause hospitalization of CKD Patient (**CKD patients were categorized into different groups according to the CAC score (0, no risk of cardiovascular events or mortality; 1-100, low risk; 101–400, intermediate risk; > 400, high risk). The survival curves for all-cause hospitalization of patients at each group were generated by the Kaplan-Meier method.)

**Fig. 4:**
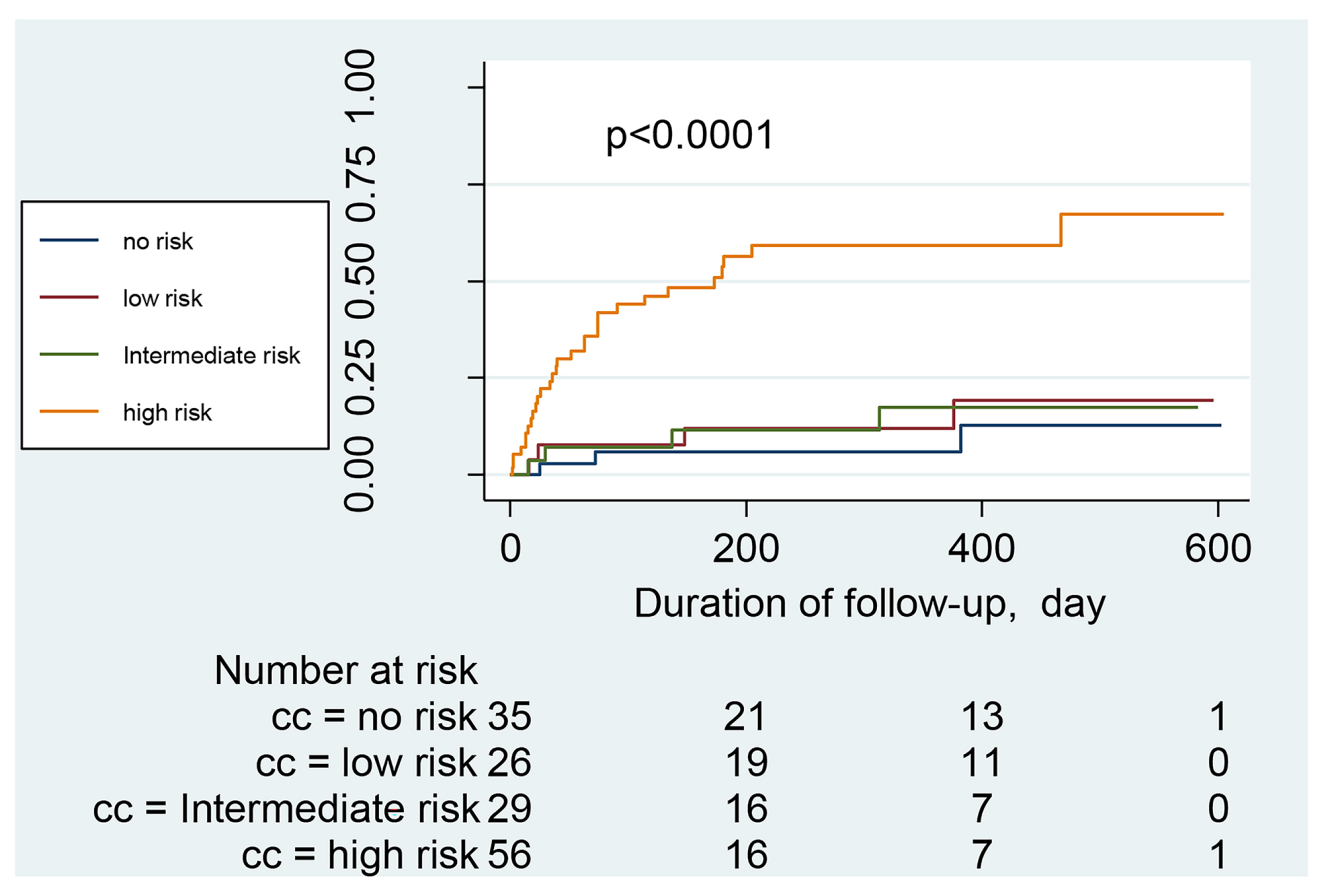
**Survival curves for cardiovascular hospitalization of CKD Patient** (CKD patients were divided into different groups based on their CAC score (0, no risk of cardiovascular events or mortality; 1-100, low risk; 101–400, intermediate risk; > 400, high risk). The survival curves for cardiovascular hospitalization of patients at each group were constructed by the Kaplan-Meier method.)

**Fig. 5:**
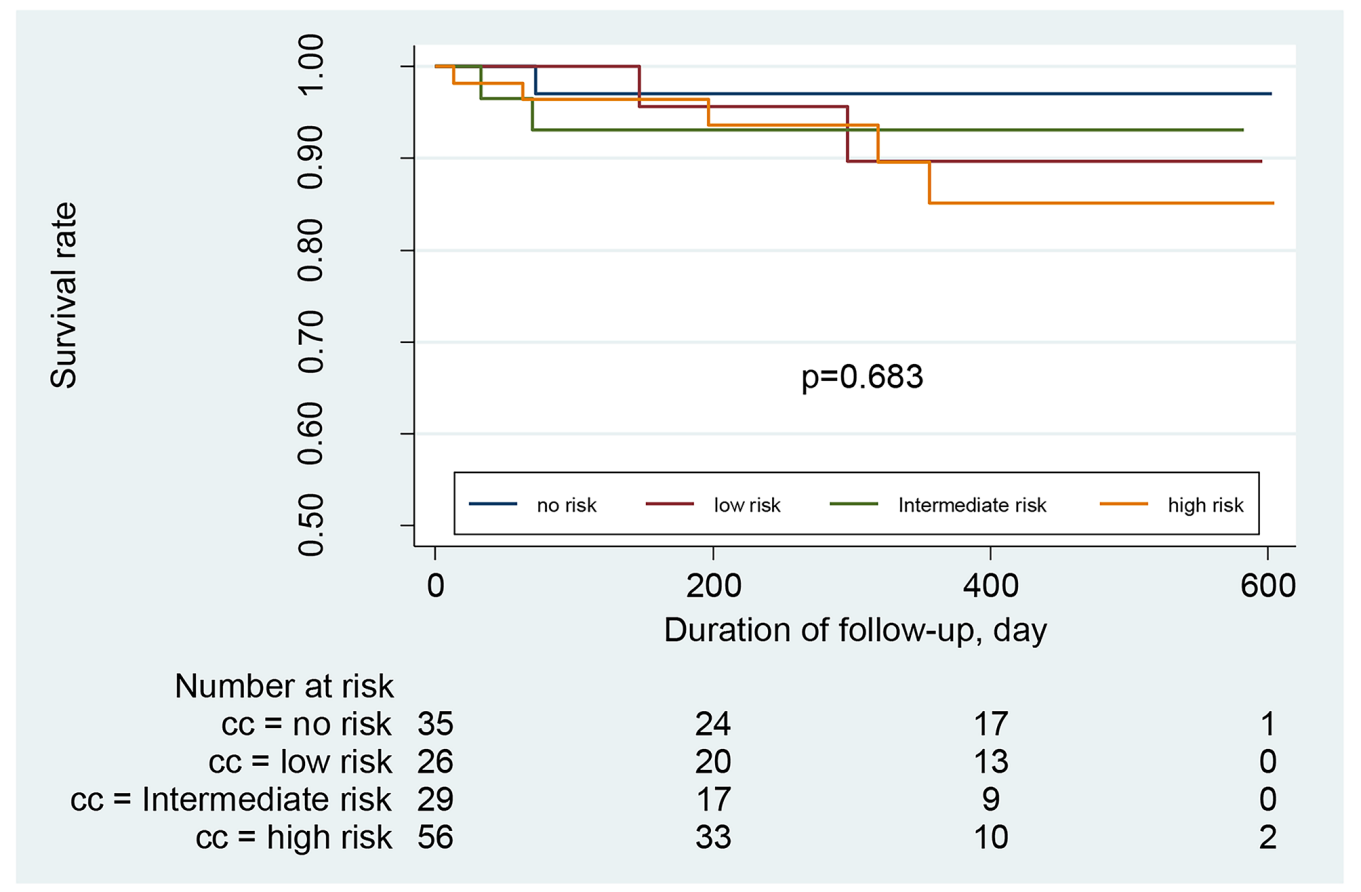
**Kaplan-Meier survival curves of CKD Patient with different CAC scores** (CKD patients were categorized into different groups according to the CAC score (0, no risk of cardiovascular events or mortality; 1-100, low risk; 101–400, intermediate risk; > 400, high risk). The Kaplan-Meier survival curves were generated using death as the endpoint of interest.)

The hospitalization and mortality rates of the patients between the administration of the CAC test and the day of data collection (or death) were recorded. The median duration was 308 days (range 34–605). During this period, 27 patients were hospitalized because of cardiovascular events. Ten patients died, with an all-cause mortality rate of 6.58% and a cardiovascular mortality rate of 4.11%. After dividing the participants into different groups according to CAC scores (no risk, low risk, intermediate risk, and high risk), we constructed Kaplan–Meier survival curves for each group using all-cause hospitalization, cardiovascular hospitalization, or death as the endpoint of interest. Patients with CAC scores of > 400 (high-risk group) had a significantly higher risk of all-cause (Fig. 3) and cardiovascular (Fig. 4) hospitalizations than patients with scores ≤ 400 (*P* < 0.0001). However, no statistically significant difference was found in the survival of patients with CKD with different CAC scores (*P* = 0.28) (Fig. 5). COX regression analysis was performed separately for all-cause hospitalization, cardiovascular hospitalization, and mortality outcomes, and CAC > 101 was found to be an independent risk factor in all-cause hospitalization (Table 3 ); it was independently associated with Age, ALB, and Urea in patients who died (Table 4); and CAC > 101 was a significant independent risk factor in patients with cardiovascular hospitalization (Table 5).

**Table 3:** Univariate and Multivariate predictor analyses of Rehospitalization

	Univariate analysis		Multivariate analysis	
	**HR(95%CI)**	**P**	**HR(95%CI)**	**P**
Age	1.02(1.01,1.03)	0.001		
ALB	0.96(0.92,0.99)	0.021	0.947(0.909,0.987)	0.009
UC	0.999(0.997,1.00)	0.100		
25[OH]D	0.978(0.96,0.997)	0.021		
CACstage*	2.31(1.52,3.52)	0.000	2.408(1.580,3.670)	0.000

*25[OH]D:25 hydroxyl-vitamin D. (CGN: DN; HTN; iPTH; β-CTx; N-MID; P1NP: LA; RV; EF.CVD; Sex; CACstage; primary renal disease; CKD stage; RRT; WBC; RGB; PLT; CRP; ALP; GLU; Urea; Scr; Ca; P; Ca*P; TC; TG; HDL; LDL; AO) P > 0.1

**Table 4:** Univariate and Multivariate predictor analyses of death

	Univariate analysis		Multivariate analysis	
	**HR(95%CI)**	**P**	**HR(95%CI)**	**P**
Age	1.10(1.04,1.17)	0.002	1.091(1.024,1.164)	0.008
CVD*	4.53(1.17,17.51)	0.029		
WBC	1.35(1.13,1.63)	0.001	1.477(1.179,1.851)	0.001
ALB	0.89(0.80,0.997)	0.044		
GLU	1.13(0.98,1.29)	0.092		
Urea	0.87(0.77,0.97)	0.013	0.845(0.755,0.945)	0.003
Scr	0.997(0.994,1.00)	0.024		
UC	0.995(0.99,1.00)	0.070		
P	0.25(0.07,0.91)	0.036		
Ca*P	0.62(0.37,1.05)	0.075		

*(CGN; DN; HTN; HD; PD; iPTH; β-CTx; N-MID; 25[OH]D; P1NP; LA; RV; EF.CVD; Sex; CACstage; CKD stage; RRT; Sex; RBC; HGB; PLT; CRP; Ca; TC; TG; HDL; LDL; AO; EF; ALP) p > 0.1

**Table 5:** Univariate and Multivariate predictor analyses of Rehospitalization -CVD

Univariate analysis			Multivariate analysis	
	**HR(95%CI)**	**P**	**HR(95%CI)**	**P**
Age	1.03(1.01,1.05)	0.013		
Sex*	1.81(0.98,3.34)	0.058		
CVD*	1.71(0.92,3.15)	0.088		
PLT	1.00(1.00,1.01)	0.022	1.004(1.001,1.008)	0.023
Scr	0.999(0.988,1.00)	0.093		
UC	0.997(0.995,1.00)	0.053		
P	0.65(0.39,1.08)	0.096		
N-MID	0.997(0.994,1.00)	0.044	0.997(0.994,0.999)	0.020
25[OH]D	0.97(0.94,1.00)	0.052		
CACstage*	4.69(2.07,10.60)	0.000	4.652(2.055,10.533)	0.000

*25[OH]D:25 hydroxyl-vitamin D. (CGN; DN; HTN; iPTH; β-CTx; N-MID; P1NP; LA; RV; EF.CVD; Sex; CACstage; primary renal disease; CKD stage; RRT; WBC; RBC; HGB; CRP; ALB; ALP; GLU; Urea; Ca; ca*p; TC; TG; HDL; LDL; AO; EF) p > 0.1

## Discussion

Vascular calcification is associated with significantly poor prognosis and mortality in patients with CKD [5]. The accumulation of Ca deposits in coronary vessels involves numerous mechanisms related to disturbances in mineral metabolism [27]. In the current study, we examined vascular calcification in patients with CKD using spiral CT and the Agatston scoring method. Our results showed that the CAC in this population was positively correlated with the serum levels of CRP, PTH, N-MID, P1NP, and β-CTx, but not Ca or P level. No significant difference was observed in the CAC scores of patients with CKD at different stages or receiving different treatments. Hospitalization analysis showed that patients with CAC scores of > 400 (indicating a high risk of cardiovascular events) had a significantly higher risk of all-cause and cardiovascular hospitalizations than those with CAC scores of ≤ 400.

Previous data showed that glomerulonephritis is the leading cause of CKD in China, followed by diabetic kidney disease, which is consistent with our results showing that chronic glomerulonephritis (39.0%) and diabetic nephropathy (28.1%) accounted for approximately 70% of all cases. The KDIGO workgroup recommended CKD-MBD, which encompasses systemic disorders of mineral and bone metabolism due to CKD, including altered levels of Ca, P, and PTH; disturbed bone modeling; and calcification in arteries and soft tissues [28]. Patients with CKD undergoing dialysis often demonstrate CKD-MBD-related symptoms, such as bone abnormalities and arterial calcification, making them high-risk for cardiovascular events and high morbidity and mortality rates [29]. In our study, the prevalence of CAC was 58.22% in the studied population, and 56 (38.36%) of the patients had CAC scores of > 400, which was consistent with the data published in a recent meta-analysis showing that the overall prevalence of vascular calcification among patients with CKD was 60% (95% confidence interval: 53–68%) [30]. According to the Clinical Practice Guideline for CKD-MBD, the serum levels of Ca, P, and PTH in CKD stages 3–5 are recommended to be maintained at a range of 2.1–2.5 mmol/L, 0.87–1.45 mmol/L, and 150–300 pg/ml, respectively. Here, we report that only 62.65%, 55.42%, and 21.69% of the patients showed normal serum levels of Ca, P, and PTH, respectively, indicating that better management of CKD-MBD is needed for patients with CKD.

Vascular calcification is a chronic and complex pathological process that involves multiple factors. We found no significant correlation between CAC score and FBG or LV ejection fraction, which was measured only once for all patients. Inflammation plays an important role in the development of vascular calcification and is positively correlated with aortic lesions in patients with CKD [31]. CRP is an inflammatory biomarker associated with arterial calcification and atherosclerosis and has been considered an independent risk factor for cardiovascular events [32, 33]. Here, we report a significant and positive correlation between CRP levels and CAC scores in patients with CKD.

Hyperphosphatemia and hypercalcemia are key factors that contribute to the initiation and progression of arterial calcification in patients with CKD [34]. Previous evidence revealed that serum Ca–P product is an independent risk factor for vascular calcification in patients with CKD undergoing hemodialysis [35]. In this study, we found no significant correlation between CAC score and serum Ca or P. As these minerals are also affected by diet, nutritional status, or medications, a single measurement may not necessarily reflect the whole-body Ca and P.

An increase in serum PTH level has been reported to stimulate the release of Ca and P from bones, resulting in mineral metabolic disorders and vascular calcification in patients with CKD [36]. Consistently, our analysis showed a significant positive correlation between CAC score and PTH level. Patients with CKD often present with vitamin D deficiency and low levels of 25(OH)D, the predominant circulating form of vitamin D. A decrease in 25(OH)D level leads to reduced intestinal Ca absorption, decreased total Ca level, and elevated PTH level in the blood [37]. A study of 289 hemodialysis patients showed that low 25(OH)D levels are inversely related to vascular calcification, but the statistical significance is lost after correcting for confounding factors [38]. In our pooled population, no significant correlation was observed between CAC score and 25(OH)D level. As some patients were administered bioactive vitamin D (calcitriol) during the study, the 25(OH)D level could have been altered, thereby reducing the risk of vascular calcification.

A relationship between vascular calcification and low bone turnover has been reported in hemodialysis patients [39]. In addition, the co-incidence of arterial calcification and osteoporosis implies that the correction of the imbalance in bone turnover may protect patients with CKD against vascular calcification [40]. In the current study, we analyzed bone metabolism parameters, including P1NP, β-CTx, and N-MID, to determine the correlations between CAC score and osteogenic activity, bone degradation, and bone turnover ratio. The results showed that CAC was positively correlated with P1NP, β-CTx, and N-MID levels in patients with CKD, suggesting the clinical value of bone turnover markers for predicting vascular calcification in these patients. Further hospitalization and survival analysis showed that patients with CAC scores of > 400 had a significantly higher risk of all-cause and cardiovascular hospitalizations than patients with scores of ≤ 400. Despite differences in the survival curves of patients with CKD with different CAC scores, no statistical significance was observed, possibly because of the short duration of observation and the small sample size. Future investigations with a multi-center, prospective design, and larger sample size with non-hospitalized patients with CKD are warranted to validate the results of the current study and further explore the mechanisms of vascular calcification in CKD.

## Conclusion

The main cause of CKD was glomerulonephritis, followed by diabetic kidney disease. The CAC score was positively correlated with markers of inflammation and bone metabolism. Patients with CKD with CAC scores of > 400 showed a higher risk of all-cause and cardiovascular hospitalizations than those with low CAC scores. Therefore, a balanced bone metabolism is essential for the maintenance of cardiovascular health in patients with CKD.

## Data Availability

The data that support the findings of this study are not publicly available due to their containing information that could compromise the privacy of research participants. But are available from the corresponding author on reasonable request; Dr Yueming Liu (lyman6136@126.com).
